# *Moringa oleifera* Lam. Peptide Remodels Intestinal Mucosal Barrier by Inhibiting JAK-STAT Activation and Modulating Gut Microbiota in Colitis

**DOI:** 10.3389/fimmu.2022.924178

**Published:** 2022-07-15

**Authors:** Zi-Shan Hong, Jing Xie, Xue-Feng Wang, Jing-Jing Dai, Jia-Ying Mao, Yu-Ying Bai, Jun Sheng, Yang Tian

**Affiliations:** ^1^ College of Food Science and Technology, Yunnan Agricultural University, Kunming, China; ^2^ National Research and Development Professional Center for Moringa Processing Technology, Yunnan Agricultural University, Kunming, China; ^3^ Engineering Research Center of Development and Utilization of Food and Drug Homologous Resources, Ministry of Education, Yunnan Agricultural University, Kunming, China; ^4^ Yunnan Provincial Engineering Research Center for Edible and Medicinal Homologous Functional Food, Yunnan Agricultural University, Kunming, China; ^5^ Key Laboratory of Pu-er Tea Science, Ministry of Education, Yunnan Agricultural University, Kunming, China

**Keywords:** *Moringa oleifera* seed, peptide, colitis, intestinal mucosal barrier, gut microbiota, JAK-STAT pathway

## Abstract

Ulcerative colitis is a chronic inflammatory bowel disease (IBD), but progress in exploring its pathogenesis and finding effective drugs for its prevention and treatment has stalled in recent years. The seeds of *Moringa oleifera* Lam. are rich in proteins known to have multiple physiological activities. In our earlier work, we had isolated and purified a peptide (MOP) having the sequence KETTTIVR, from *M. oleifera* seeds; however, its anti-inflammatory activity and mechanism *in vivo* were unclear. Here we used the dextran sulfate sodium (DSS)-induced colitis model to study the anti-inflammatory activity and mechanism of this MOP. Our results are the first to show that MOP can ameliorate the pathological phenotype, inflammation, and intestinal barrier disruption in mice with colitis. Furthermore, RNA sequencing revealed that MOP inhibits the Janus kinase/signal transducer and activator of transcription (JAK-STAT) pathway activation. Next, by using 16s rRNA gene sequencing, we found that MOP can ameliorate DSS-induced gut microbiota dysbiosis. In addition, an untargeted metabolomics analysis suggested that MOP is able to modulate the level of lipid and amino acid metabolites in IBD-stricken mice. Altogether, these results indicate that MOP ameliorates colitis by remodeling intestinal mucosal barrier by inhibiting JAK-STAT pathway’s activation and regulating gut microbiota and its metabolites, thus providing a basis for further processing and design of bioactive foods from *M. oleifera* seeds.

## Introduction

Inflammatory bowel disease (IBD) is a chronic inflammatory disease of the intestines, including Crohn’s disease (CD) and ulcerative colitis (UC) (1, 2). Worryingly, in recent years the prevalence and incidence of IBD have both increased annually (1, 3). In addition, patients who have had IBD for many years face higher risks of developing related cancers, which can adversely affect their body and mind (4, 5). Traditional drugs for treating IBD, including 5-aminosalicylic acid (5-ASA), glucocorticoids or immunomodulators, can relieve its progression but these drugs may have significant side effects (6). Therefore, it is imperative to explore natural products and their derivatives as support for the treatment and management of IBD.

The intestinal mucosal barrier consists of mechanical barrier, chemical barrier, microbial barrier and immune barrier (7). Notably, the integrity of physical barriers in the gut is regulated in part by cytokines produced by gut-resident cell (8, 9). Those cytokines that play key roles in IBD exert biological effects by inducing activation of Janus kinase/signal transducer and activator of transcription (JAK-STAT) by binding to cytokine receptor (10, 11). Moreover, there is growing evidence suggests that an imbalance between cytokines and excessive activation of the JAK-STAT pathway leads to gut barrier disruption, disease perpetuation and tissue destruction (8, 12). Therefore, inhibiting activation of the JAK-STAT pathway may be an effective way to impair intestinal inflammation and improve the intestinal barrier.

Gut microbiota is one of the major components of the intestinal mucosal barrier (13, 14). A large body of evidence suggests that changes in the composition and function of gut microbes are key factors directly contributing to the onset and progression of IBD (15), and therefore targeting the gut microbiota may be a novel strategy for the treatment of IBD. Furthermore, bioactive metabolites derived from host and gut microbiota, such as short-chain fatty acids, amino acids, choline derivatives, and indole derivatives, are important molecules that orchestrate the interaction between gut microbiota and host medium (16, 17). Notably, these metabolites have been reported to play an indirect role in remodeling the intestinal barrier by modulating signal transduction and immune responses (18).

*Moringa oleifera* Lam., in the plant family Moringaceae, has various pharmacological potential and health benefits (19, 20). In particular, studies have shown that *M. oleifera* seeds are rich in protein and harbor a variety of therapeutic properties, namely antibacterial, anti-oxidative stress, anti-inflammatory, and anti-cancer activities (21–23). Although much research has investigated the biological activity of *M. oleifera* seeds, far fewer studies have considered the proteins in these seeds, and even less is known about their peptides. According to many studies, peptides can have immunomodulatory effects and may ameliorate colitis injury by reducing inflammatory cell infiltration, improving intestinal barrier, and modulating host gut microbiota (24, 25). Currently, it is unclear whether *M. oleifera* seed-derived peptides are also anti-inflammatory, and if so, whether they modulate gut microbiota and regulate metabolism *in vivo* to exert their anti-inflammatory effects *in vivo*, especially vis-à-vis acute colitis. In earlier work, we had identified the ultrafiltration peptide components (i.e., < 3 kDa) of *M. oleifera* seeds protein hydrolysates, finding a highly active α-glucosidase inhibitory peptide with the amino acid sequence KETTTIVR that also exhibited anti-inflammatory activity *in vitro (* 26). Yet whether it has anti-UC activity *in vivo* and its mechanism of action remain unclear.

In this study, we demonstrate, for the first time to our best knowledge, the beneficial effects of an active peptide (KETTTIVR, MOP) identified by our team from *M. oleifera* seeds in mice with ulcerative colitis and explored the mechanisms by it ameliorates colitis.

## Results

### MOP Improved the Pathological Phenotype of DSS-Induced Colitis

**Figure 1:**
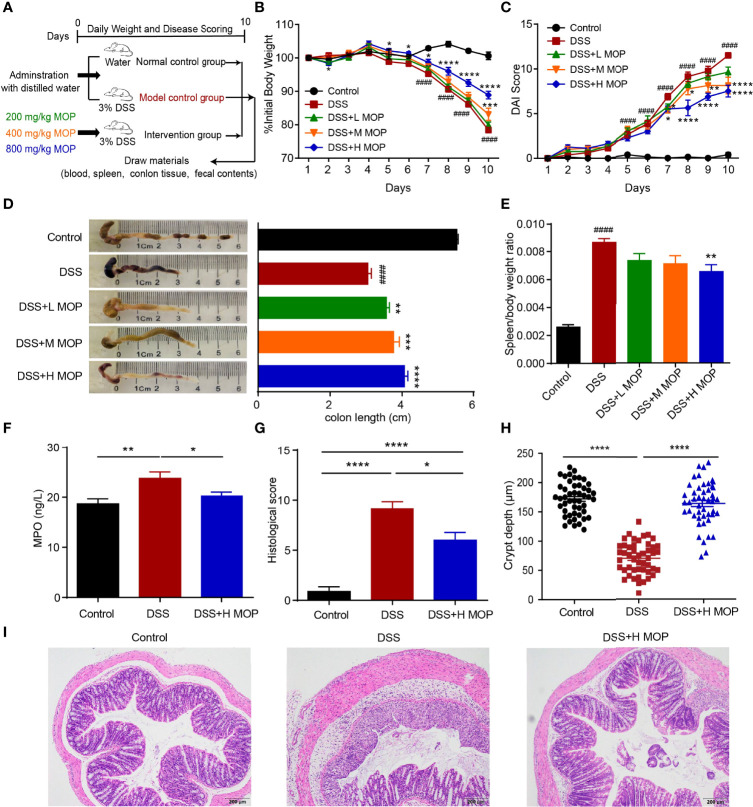
MOP ameliorated the pathological phenotype of DSS-induced colitis. **(A)** Study design of the *in vivo* mouse experiment. Colitis was induced by administration of 3% DSS dissolved in drinking water for 10 days. **(B)** Daily changes in body weight in each experimental group (n = 8) during disease progression. **(C)** Daily assessment of DAI scores in each group. **(D)** Colons were observed and the colon length was determined fo each group (n = 8). **(E)** Calculation of the spleen-to-body weight ratio (n = 8). *P < 0.05, **P < 0.01, ***P < 0.001, and ****P < 0.0001 *vs*. DSS; ^#^P < 0.05, ^##^P < 0.01, ^###^P < 0.001 and ^####^P < 0.0001 *vs*. control. **(F)** Measurement of MPO activity in serum (n = 8). **(G)** Histological scoring and **(I)** representative images of HE-stained colonic sections (scale bar = 200 μm, n=8). **(H)** Quantification of crypt depth in the colon (n = 48). Data are the mean ± SEM. *P< 0.05, ** P< 0.01, ***P < 0.001 and ****P < 0.0001. Statistical analysis was performed using Student’s t-test.

To investigate the effect of the MOP on dextran sulfate sodium (DSS)-induced colitis, colitis was induced in mice by continuous administration of 3% DSS in water for 10 days, with different doses of MOP supplemented to them during DSS induction (**Figure 1A**). Compared with the DSS group, DSS-induced colitis was significantly reduced in DSS+H MOP group, as evinced by their significant weight loss (**Figure 1B**), reduced DAI scores (**Figure 1C**), and remission of colonic shortening (**Figure 1D**). Moreover, an enlarged spleen was observed in colitis mice whereas the MOP treatment was capable of significantly relieving that splenomegaly condition, particularly in the high dosage group (**Figure 1E**). We next examined the level of myeloperoxidase (MPO) in serum, finding it was significantly higher in DSS-induced colitis mice than the control group, whereas the high dosage of MOP intervention reversed this abnormal change (**Figure 1F**). Histological analysis uncovered colonic mucosal damage in colitis mice. Importantly, the high dosage of MOP intervention significantly reduced such mucosal damage (including the greater crypt depth) and inflammatory cell infiltration, resulting in lower histological scores (**Figures 1G–I**). These results suggested the MOP treatment significantly improved DSS-induced colitis.

### MOP Improved the Colon Inflammation and Gut Barrier Disruption of DSS-Induced Colitis

**Figure 2:**
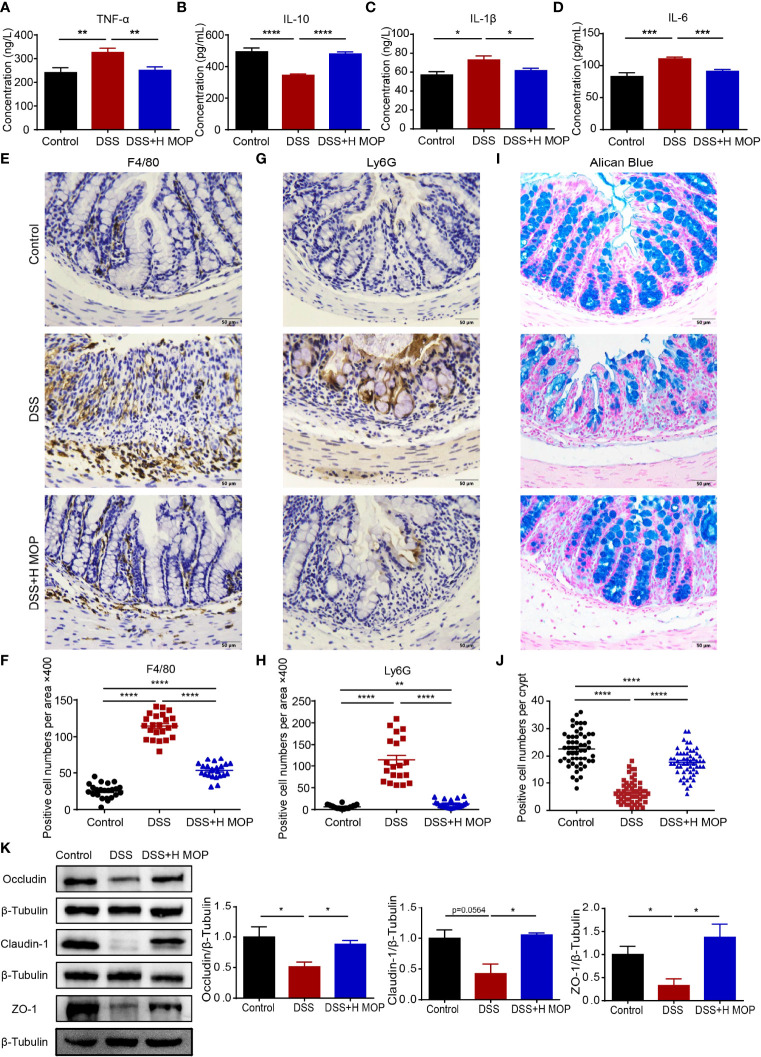
MOP ameliorates colonic inflammation and intestinal barrier disruption in DSS-induced colitis. **(A–D)** Expression levels of TNF-α, IL-6, IL-1β, and IL-10 in serum determined by ELISA (n = 8); **(E, F)** Representative immunohistochemical images of macrophages (F4/80) in mouse colon sections (scale bar = 50 μm, n=8) and the number of positive cells (n = 23); **(G, H)** Representative images of immunohistochemistry of neutrophils (Ly6G) in mouse colon sections (scale bar = 50 μm, n=8) and the number of positive cells (n = 20). **(I, J)** Representative images of Alcian blue-stained colon sections (scale bar = 50 μm, n=8) and the number of mucus-producing cupped cells (n = 40). **(K)** Representative images of ZO-1 and occluding, and representative protein blot images of claudin-1, with their associated protein expression normalized to β-tubulin. Quantification of immunoblots, performed in Image J software (n = 3). Data are the mean ± SEM. *P < 0.05, **P < 0.01, ***P < 0.001, and ****P < 0.0001. Statistical analysis was performed using Student’s t-test.

To further estimate the influence of MOP upon inflammation in colitis, we detected the levels of TNF-α, IL-1β, IL-6, and IL-10 in the serum from the control, DSS, and DSS + H MOP groups (**Figures 2A–D**). Higher levels of pro-inflammatory cytokines including TNF-α, IL-1β, and IL-6 and lower levels of anti-inflammatory cytokine IL-10 were found in colitis mice when compared with the control group. By contrast, MOP reversed these changes to cytokines in serum. Consistently, we found that MOP decreased the expression level of *TNF-α*, *IFN-γ*, *IL-1β*, and *IL-6* but increased that of *IL-10* in colon tissue (**Supplementary Figures 2A–E**).

To assess how MOP may affect the DSS-induced colonic inflammatory infiltration, colon tissue sections were stained for specific markers of macrophages (F4/80) and neutrophils (LY6G). We found that the DSS group displayed macrophage and neutrophil infiltration, as evinced by its significantly increased counts of F4/80 and Ly6G-positive cells. Conversely, MOP intervention evidently reduced the infiltration of macrophages and neutrophils (**Figures 2E–H**).

Furthermore, alisin blue staining results revealed fewer mucus-secreting goblet cells in the DSS group than the control group. In stark contrast, the MOP intervention reversed this the reduction of goblet cells as well as damage to the colon crypt (**Figures 2I, J**). In line with these findings, the expression level for the gene encoding mucin-2 (*Muc-2*) in colon tissue showed the same trend (**Supplementary Figure 2F**). To evaluate the expression of antibacterial peptides in colitis, we examined the expression levels of antimicrobial peptide genes in colon tissue, finding that the MOP treatment reversed the DSS-induced decrease in mRNA expression levels of both *Reg3b* and *Reg3g* (**Supplementary Figures 2G, H**).

To further study MOP’s effect on the gut barrier, we examined the mRNA expression levels of zonula occludens 1 (ZO-1), occludin, and claudin-1 in mice colon tissue. Their expression levels were significantly lower in the colitis group than the control group. But the MOP administration significantly reversed the DSS-induced downregulation in the levels of *occludin*, *ZO-1*, and *claudin-1* (**Supplementary Figures 2I–K**). To confirm this pattern in gene expression, we used western blotting to assess the protein expression of ZO-1, occludin, and claudin-1; these results were consistent with those of their genes’ mRNA expression (**Figure 2K**). Collectively, these results suggested that MOP treatment suppressed DSS-induced colonic inflammation and attenuated the DSS-impaired functioning of the intestinal mucosal barrier.

### MOP Ameliorates Colitis by Inhibiting Activation of the JAK-STAT Pathway

**Figure 3:**
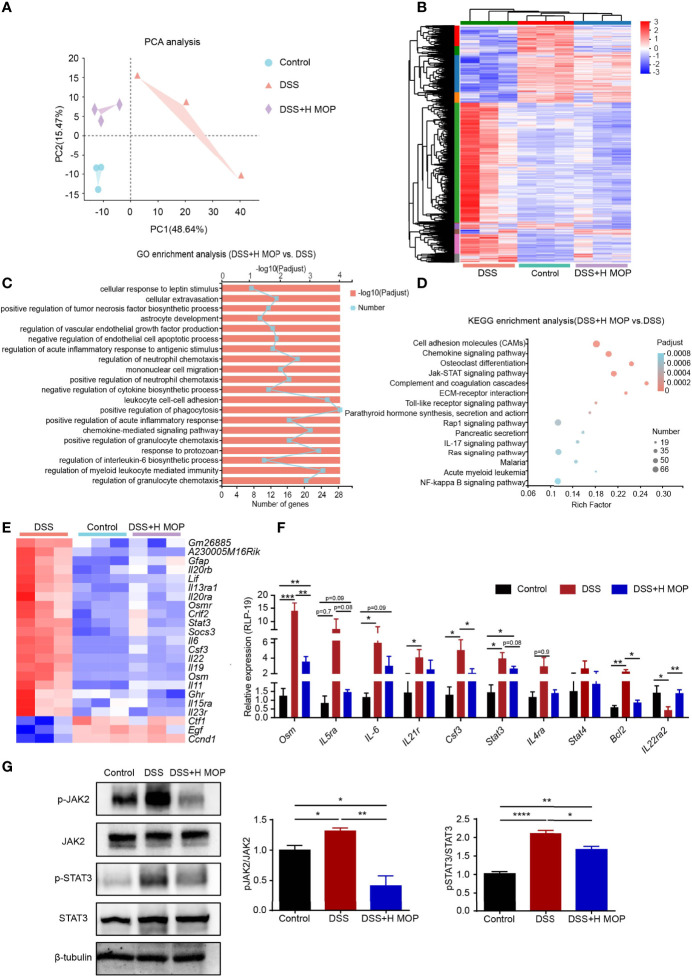
MOP regulates colonic intestinal function and inhibits the JAK-STAT pathway. **(A)** Principal component analysis (PCA) between the three groups. **(B)** Heatmap of three groups of DEGs (differentially expressed genes). The color in the graph indicates the expression value of the gene after normalization in each sample, with red indicating higher expression of a given gene in that sample and blue indicating its lower expression. **(C)** GO enrichment analysis of DEGs between the DSS and DSS+H MOP groups. **(D)** KEGG enrichment analysis of DEGs between the DSS and DSS+H MOP groups. **(E)** Heatmap of DEGs in the JAK-STAT pathway. **(F)** Detection of DEGs in the JAK-STAT pathway by RT-qPCR (n = 6). **(G)** Representative western blotting images for the expression and phosphorylation of JAK2 and STAT3, whose relative protein expression levels were quantified and normalized to β-tubulin (n = 3). Data are mean ± SEM. *P < 0.05, **P < 0.01, ***P < 0.001 and ****P < 0.0001. Statistical analysis was performed using Student’s t-test.

To determine the underlying biological processes and pathways by which MOP ameliorates DSS-induced colitis in mice, RNA-Seq analyses were conducted. Significant differences in transcriptional profiles were detected among control, DSS, and DSS+H MOP mice groups (**Figures 3A, B**). Specifically, relative to the control group, DSS caused the upregulation of 2274 genes and the downregulation of 1280 genes in the mouse colon (**Supplementary Figures 3A, B**). In addition, a total of 621 and 1873 genes were respectively uregulated and downregulated in the DSS+H MOP group compared with the DSS group (**Supplementary Figure 3C**). The follow-up GO enrichment analysis indicated that MOP modulates the immune response of DSS-induced colitis (**Figure 3C**). Interestingly, the KEGG pathway analysis of the differentially expressed genes (DEGs) indicated that the JAK-STAT pathway was a highly enriched functional pathway (**Figure 3D**). Heatmap results showed that DSS induced the upregulation of JAK-STAT pathway-related genes’ expression in mice with colitis, but the MOP administration significantly downregulated it (**Figure 3E**). This suggested the MOP intervention could have exerted its anti-inflammatory effects by inhibiting the JAK-STAT signaling pathway.

To further confirm whether or not MOP inhibits the JAK-STAT pathway, RT-qPCR was used to check the expression levels of key genes operating in this pathway (**Figure 3F**). The results revealed augmented levels of their expression in the DSS group vis-à-vis the control group. As expected, in the DSS+H MOP group the increasing expression of these genes involved in the JAK-STAT pathway was reversed. Consistent with that, the western blotting results showed that phosphorylation levels of JAK2 and STAT3 were significantly increased in DSS-induced colitis mice, and MOP administration reversed this trend (**Figure 3G**). Overall, MOP suppressed inflammatory responses by participating in DSS-induced immunomodulation and inhibiting the JAK-STAT pathway in mice with colitis.

### MOP Could Alleviate Gut Microbiota Dysbiosis in Colitis Induced by DSS

**Figure 4:**
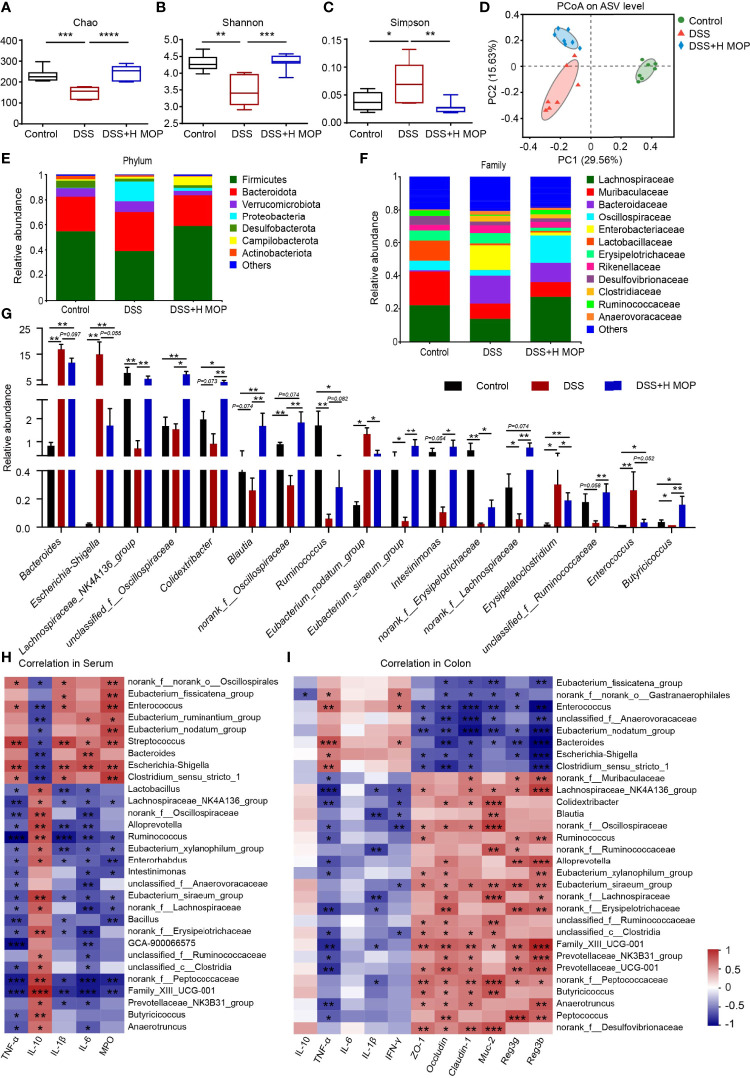
MOP modified the dysbiosis of intestinal microbiota in DSS-induced colitis. **(A–C)** Alpha diversity index analysis of intestinal microbiota abundance (Chao index) and diversity (Shannon and Simpson indexes). **(D)** Principal coordinate analysis (PCoA) plots of Bray–Curtis distance matrix for the composition of gut microbiota of different groups at the ASV level. Community structure composition of different microbial groups, at the taxonomic level of phylum **(E)** and family **(F)**. **(G)** Comparison of relative abundances at the genus level among the three experimental groups (n = 7). **(H)** Spearman correlations between gut microbiota and inflammatory factors and MPO enzyme activity in mice serum parameters (n = 7). **(I)** Spearman correlations between intestinal microbiota and parameters of inflammatory genes, intestinal barrier genes, and antimicrobial peptide genes in mouse colon tissue (n = 6). The red coloring indicates positive correlations, while the blue coloring indicates negative correlations. The intensity of the color is proportional to value (strength) of the Spearman correlation coefficient. Data are the mean ± SEM. *P < 0.05, **P < 0.01, and ***P < 0.001.

To investigate whether MOP can alleviate DSS-induced gut microbiota dysbiosis, we used 16S rRNA gene amplicon sequencing to profile the composition of mouse gut microbiota. These results showed that MOP ameliorated the DSS-induced reduction in gut microbiota richness and diversity in mice with colitis, as seen in the changed Chao, Shannon, and Simpson indices (**Figures 4A–C**). The Bray-Curtis-based PCoA (principal coordinates analysis) revealed a significant difference between the control and DSS groups (R2 = 0.393, P = 0.001, **Figure 4D**). Further, gut microbiota structures were also segregated between the DSS+H MOP and control groups (R2 = 0.3693, P = 0.001) and likewise for the DSS and DSS+H MOP groups (R2 = 0.2453, P = 0.001). Next, we performed a LEfSe analysis of the differential gut microbiota found between the three groups at the taxonomic level, from the phylum to genus (**Supplementary Figure 4**). These results indicated that *Escherichia-Shigella* (from the Proteobacteria phylum to genus), *Bacteroides* (from the Bacteroidetes family to genus), and *Clostridium_sensu_stricto_1* (from the Clostridium phylum to genus) were the dominant microbial taxa in the DSS group, while *Blautia* and *Oscillibacter* (from Firmicutes phylum to genus) were relatively enriched in the DSS+H MOP group.

To further investigate the possible impact of MOP on the gut microbiota of mice with colitis, we analyzed different taxa in the three experimental groups. At the phylum level, Firmicutes, Bacteroidetes, Verrucomicrobia, and Proteobacteria were the major phyla dwelling in the cecal contents microbiota (**Figure 4E**). At the family level, in the DSS group the relative abundance of Enterobacteriaceae increased while that of Lachnospiraceae as well as Oscillospiraceae decreased, being characteristic of gut dysbiosis that could be reversed by the MOP treatment (**Figure 4F**). The changed microbiota at the genus level are depicted in **Figure 4G**. Compared with the control group, the relative abundances of *Bacteroides*, *Escherichia-Shigella*, and *Enterococcus* increases in colitis mice. However, MOP treatment restored the relative abundances of these various bacteria. It is crucial to note that MOP caused an enrichment of *Lachnospiraceae_NK4A136_group*, *norank_f:Lachnospiraceae*, *Blautia*, *Ruminococcus*, and *unclassified_f:Ruminococcaceae*, these belonging to Lachnospiraceae and Ruminococcaceae, respectively.

To understand the associations between differentially enriched microbes and inflammatory factors, intestinal barrier, and antimicrobial peptides, Spearman correlations were performed (**Figures 4H, I**). These results showed that *Eubacterium_fissicatena_group*, *Enterococcus*, *Eubacterium_nodatum_group*, *Bacteroides*, *Escherichia-Shigella*, and *Clostridium_sensu_stricto_1* each had a strong positive correlation with the TNF-α, IL-1β, IL-6, and MPO levels in serum, as well as the TNF-α level in colon. However, those six taxa had negative correlations with the mRNA IL-10 levels (P < 0.05) in serum, and likewise with colonic barrier proteins ZO-1, occludin (P < 0.01), claudin-1 (P < 0.001), mucin Muc-2, and antimicrobial peptides Reg3g, Reg3b (P < 0.01) levels in the colon, respectively. In addition, *Lachnospiraceae_NK4A136_group*, *Ruminococcus*, *norank_f:Lachnospiraceae*, *unclassified_f:Ruminococcaceae*, and *Family_XIII_UCG-001*, were negatively correlated with TNF-α, IL-1β, IL-6, and MPO levels in the serum, yet positively correlated with IL-10 in the serum, *ZO-1*, *occludin* and *claudin-1*, *Muc-2*, and *Reg3g*, *Reg3b* in the colon. Altogether, these results suggested that MOP can alleviate DSS-induced gut microbiota dysbiosis and increased the relative abundance of Lachnospiraceae and Ruminococcaceae members in particular.

### MOP Alters Metabolic Profiles and Regulates the Metabolism of Lipids and Amino Acids

**Figure 5:**
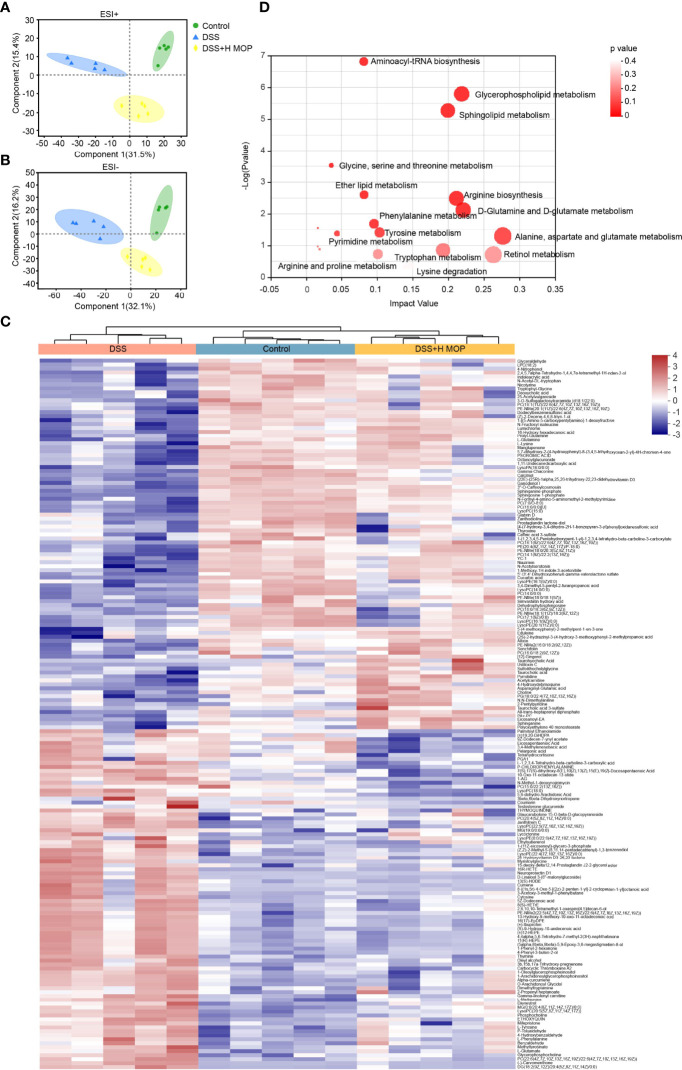
MOP alters metabolic profiles and regulates the metabolism of lipids and amino acids. **(A, B)** Linear discriminant analysis of metabolomic profiles in mouse serum in the positive ion **(A)** and negative ion **(B)** mode (n = 5). **(C)** Heatmap of significantly altered metabolites. **(D)** KEGG pathway enrichment analysis of differential metabolites.

To investigate the effect of MOP on the metabolic profile of colitis mice, we analyzed plasma samples *via* untargeted metabolomics. The resulting PLS_DA plot showed that the metabolic profiles of DSS-induced colitis mice and the control group differed significantly, in terms of both negative and positive ion mode, while the MOP intervention group was also significantly different from the DSS group (**Figures 5A, B**). The resulting PCA plot also indicated a satisfactory classification among the three groups (**Supplementary Figures 5A, B**). We detected 325 different metabolites between the three groups of mice (P < 0.05, **Supplementary Table S1**). According to the heatmap analysis of significantly changed differential metabolites (**Figure 5C**), phosphatidylcholine (PC; 18:1/22:6, 14:0/0:0, 16:0/18:3, 16:1/22:6), lysophosphatidylcholine (LPC; 18:2, 16:0), and LysoPC (15:0, 16:1/0:0) in the DSS group—as previously reported by Tefas et al. (27)—being significantly lower than the control group; however, the MOP treatment significantly reversed the trend of decreasing these metabolites, which is striking. Using the KEGG database, the differential metabolites were then subjected to pathway enrichment analysis; 15 significantly enriched metabolic pathways (P < 0.05) were identified, these mainly involving amino acid and lipid metabolism (**Figure 5D**). To explore the relationship of metabolites to inflammatory factors and gut barrier and antimicrobial peptides, the top-100 differential metabolites in terms of their relative abundance underwent Spearman correlations (**Supplementary Figure 5C**). These metabolites had strong correlations with inflammatory factors (TNF-α, IL-1β, IL-6, IL-10, IFN-γ) and MPO levels in serum, and intestinal barrier (*ZO-1, occludin, claudin-1*), mucin (*Muc-2*) and antimicrobial peptides (*Reg3b, Reg3g*) mRNA levels in colon. We used Spearman correlation analysis to identify potential relationships between alterations in gut microbes and metabolites (**Supplementary Figure 5D**). Our findings reveal a significant correlation between gut microbes and metabolites.

**Figure 6:**
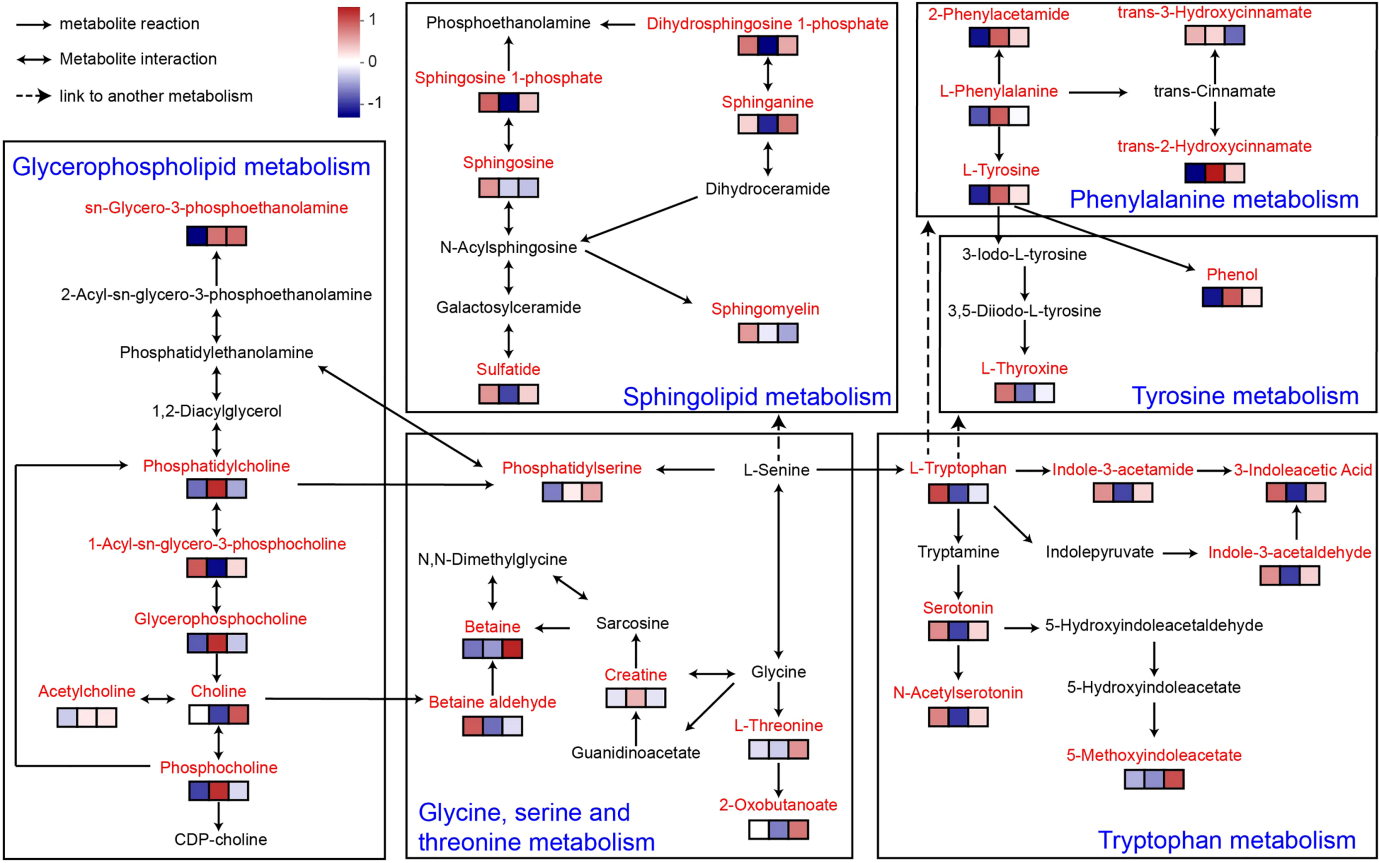
Metabolic network of MOP-treated mice with colitis. Enriched metabolites are marked in red. The expression of metabolites in the different experimental groups is conveyed by the heatmap blocks, based on Euclidean analysis, corresponding from left to right to the control, DSS, and DSS+H MOP groups. The differing colors denote the magnitude of metabolites’ relative expression in the samples of a given group, with redder colors indicating the higher expression of metabolites and bluer colors indicating the lower expression of metabolites (n = 5).

According to the KEGG database/pathway enrichment results, the significantly enriched plasma metabolites and metabolic pathways were interconnected and formed a discernible metabolic network (**Figure 6**). The metabolic pathways involved include sphingolipid metabolism, sphingolipid metabolism, glycine, serine and threonine metabolism, tyrosine metabolism, tryptophan metabolism, and phenylalanine metabolism. This suggested that MOP has ameliorated DSS-induced colitis not only by modulating a single metabolic pathway, but also indirectly *via* interrelationships between certain metabolites, thereby modulating a complex metabolic network.

## Discussion

**Figure 7:**
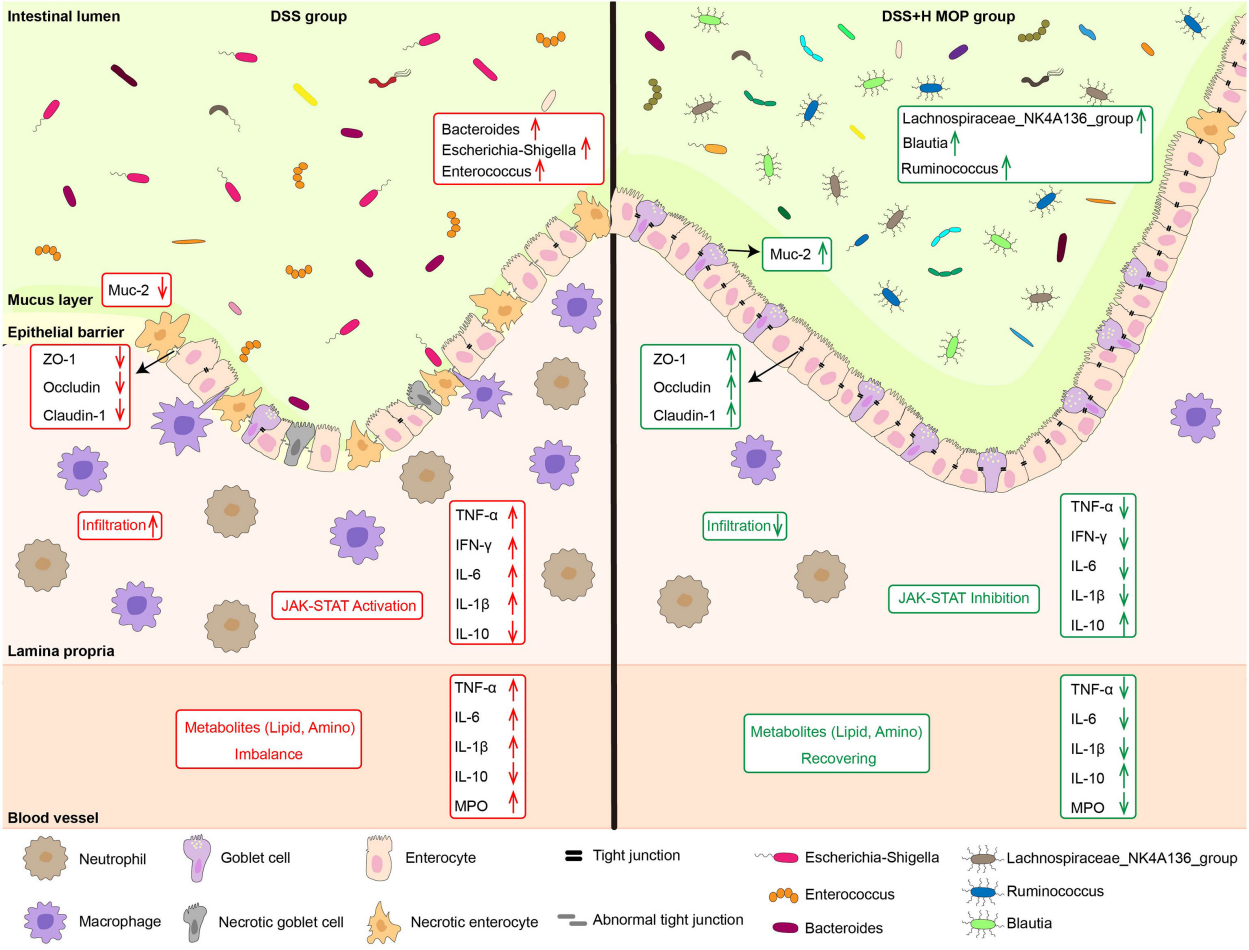
Schematic diagram of the mechanism of action of MOP in alleviating colitis. MOP ameliorated colitis by remodeling the intestinal mucosal barrier by inhibiting the activation of the JAK/STAT pathway, modulating the composition and function of the gut microbiota, as well as the levels of lipid and amino acid metabolites.

Recent epidemiological statistics indicate that the incidence of UC continues to increase worldwide, now affecting 0.3% of the global population (28). Therefore, it is particularly urgent to develop natural medicines with low side effects and proven safety. Many studies have shown that peptides have various physiological effects such as anti-inflammatory ones (29). Yet whether MOP is capable of alleviating colitis and its mechanism of action both remain unclear. In our study, we found that MOP ameliorates colitis by remodelling intestinal mucosal barrier. Its potential protective mechanism may be through inhibiting the activation of the JAK-STAT pathway, regulating the composition and function of gut microbiota, and the level of lipid and amino acid metabolites. These findings suggest that MOP holds promise as a natural and effective drug for supporting to IBD by restoring the intestinal mucosal barrier (**Figure 7**).

As a new kind of natural medicine, peptides have many advantages, such as their high bioactivity and selectivity, lower toxicity than chemical drugs, easy absorption, and reduced degree of accumulation (30). Similarly, we previously studied the *in vitro* hypoglycemic activity and stability of MOP, and found that after MOP was digested by pepsin, it still had an inhibitory effect on α-glucosidase activity, with an inhibition rate of 97.60%. And after MOP was digested by trypsin, it still had inhibitory effect on α-glucosidase activity, and the inhibition rate was 86.76% (26). Through the above studies, we believe that MOP has a certain resistance to pepsin and trypsin, and has good stability. Therefore, we believe that MOP is a potential, highly exploitable, functional food for the adjuvant treatment of colitis.

Several studies have shown that many active peptides have anti-inflammatory properties and harbor potential to treat ulcerative colitis (31, 32). For example, Ala-Gln can prevent colitis through PepT1 and by decreasing the abundance of Bacteroidetes and the ratio of Bacteroidetes to Firmicutes (33). Furthermore, food protein-derived VPP and IPP inhibit colitis through NF-κB and MAPK pathways (34). In the present study, the MOP administration attenuated inflammation by inhibiting the JAK-STAT pathway and significantly increased the relative abundances of *Lachnospira*, *Blautia*, and *Ruminococcus*. Although our findings are consistent with previous studies, there are still many noteworthy differences, and these may be related to the peptide sequence and amino acid composition (35).

Intestinal mucosal barrier dysfunction is a key pathology of colitis (36). In the immune barrier, overproduction of pro-inflammatory cytokines (TNF-α, IFN-γ, IL-1β, and IL-6) or reduced production of anti-inflammatory cytokines (IL-10) induces the persistence and severity of IBD, worsen the intestinal environment (37, 38). In agreement with previous studies (39, 40), MOP significantly reversed DSS-induced changes in cytokine (TNF-α, IFN-γ, IL-1β, IL-6, and IL-10) levels. Notably, changes in cytokine levels are often caused by inflammatory cell infiltration (41, 42). Therefore, we examined the expression of immune cells (neutrophils and macrophages), and as expected, MOP significantly reversed the DSS-induced infiltration of F4/80+ macrophages and Ly6G+ neutrophils, which consistent with previous studies (43). Furthermore, MOP attenuated DSS-induced impairment of physical barrier function, as evidenced by enhanced tight junction expression and increased goblet cell numbers and mucus expression, consistent with previous studies (40, 44, 45). Collectively, our results suggest that MOP has anti-inflammatory effects and ameliorates DSS-induced intestinal barrier dysfunction.

It is worth noting that cytokines such as IFN-γ and IL-6 are released and bind to receptors on cells, thereby activating JAK-STAT pathway, further up-regulates claudin-2 expression, and mislocalizes ZO-1 to form intercellular spaces, thereby increasing intestinal epithelial permeability (46–48). In our study, transcriptomics revealed that JAK-STAT pathway is a highly enriched functional pathway. And it was demonstrated by western blot that MOP could inhibit JAK-STAT pathway activation. Therefore, we believe that MOP may inhibit JAK-STAT pathway activation by inhibiting the expression of inflammatory factors, and finally regulate intestinal barrier dysfunction.

Gut microbes can directly or indirectly (through their metabolites) modulate signal transduction and immune responses to remodel the intestinal epithelial barrier. It has been reported that Proteobacteria are the major contributors to IBD (49, 50), whereas Firmicutes are depleted under IBD, resulting in dystrophic gut microbiota (51). In addition, studies have shown that Proteobacteria can invade intestinal epithelial cells and aggravate intestinal inflammation by releasing endotoxin and lipopolysaccharide (LPS), affecting intestinal permeability (52). Interestingly, our findings show Proteobacteria (including *Escherichia-Shigella* and *Enterobacter* genera) as the dominant microbiota in mice with IBD. However, Lachnospiraceae and Ruminococcaceae (Firmicutes) were significantly enriched in the DSS+H MOP group, likely inhibiting the DSS-induced increase in Proteobacteria and improving overall gut dysbiosis. This is consistent with previous studies, in which decreased relative abundances of Lachnospiraceae and Ruminococcaceae were observed in colitis (43, 53, 54). As major producers of butyrate, both taxa enhance the integrity of the intestinal epithelial barrier and suppress inflammation (54–56). Furthermore, also acting as butyrate producers (57–59), *Blautia*, *Intestinimonas*, and *Butyricicoccus* also occurred at greater relative abundances in the DSS+H MOP group than the DSS group of mice.

Furthermore, as a pathogenic bacteria, *Enterococcus* is closely related to inflammation-related diseases *in vivo (* 60). It has been shown that *Enterococcus* produces metalloproteinases, which aggravate intestinal inflammation in mice by damaging epithelial cells and destroying the integrity of the intestinal barrier (61). We detected this bacterial genus increased in the colon of the IBD group, but the MOP intervention reversed this trend. Further, the correlation results showed that *Enterococcus* was significantly positively correlated with the levels of pro-inflammatory factors in serum and pro-inflammatory genes (IFN-γ) in colon, but negatively correlated with those of anti-inflammatory factor (IL-10) in serum and colonic barrier (*ZO-1, Occludin, Claudin-1*), mucin (*Muc-2*), and antimicrobial peptide (*Reg3b, Reg3g*) in colon. Also, as a butyrate-producing microorganism (62), *Lachnospiraceae_NK4A136_group* was found significantly increased in abundance after the MOP intervention, being negatively correlated with pro-inflammatory factors and significantly positively correlated with anti-inflammatory factors, intestinal barrier, mucin, and antimicrobial peptide genes. Ruminococcaceae is reportedly more closely related to the Treg/Th17 balance and to resisting DSS-induced ulcerative colitis (63). In our study, as expected, MOP reversed DSS-induced depletion of Ruminococcaceae and was inversely associated with pro-inflammatory factors in the correlations, with anti-inflammatory factors, intestinal barrier, mucin, and antimicrobial peptides genes all positively correlated to it. Taken together, these lines of evidence suggest that the MOP administration ameliorates DSS-induced gut dysbiosis, thereby increasing the abundance of anti-inflammatory-related beneficial bacteria like Lachnospiraceae and Ruminococcaceae, which may help to promote recovery from gut barrier.

As a link coordinating the interaction between gut microbes and the host, the physiologically active compounds produced by the metabolism of the host and gut microbes play an important role. In particular, choline derivatives in lipid metabolism and indole derivatives in amino acid metabolism have many physiological functions, including regulating and maintaining intestinal mucosal immune homeostasis and enhancing intestinal barrier function (18). In our study, we found that MOP significantly regulated the levels of lipid and amino acid metabolites. In fact, lipids play an important role in maintaining the integrity of the intestinal epithelium (64). A previous study revealed an important role for the *de novo* lipogenic enzyme fatty acid synthase in maintaining gut barrier function by regulating palmitoylation of Muc2 (65). Thus, disturbances in lipid metabolism disrupt intestinal barrier integrity. In lipid metabolism (**Figure 6**), L-serine generates phosphatidylserine with the help of enzymes, which then enters glycerophospholipid metabolism and undergoes a series of metabolic transformations under the action of different enzymes to generate choline-related metabolites. Choline is a metabolite produced from phosphatidylcholine by some gut microbes with phospholipase D enzymes, which plays an important role in maintaining the structural integrity of cell membranes (17, 66, 67). Studies have shown that choline supplementation helps maintain intestinal mucosal immune homeostasis and strengthens the intestinal barrier (68). Here, MOP reversed the DSS-induced disturbance of lipid metabolite levels and had a tendency to promote choline increases. Interestingly, we found that lipid metabolites including choline were significantly associated with gut microbes. Therefore, we speculate that this may be the metabolic regulation of choline by gut microbes.

In addition, in amino acid metabolism, L-serine generates L-tryptophan under the enzymatic action of tryptophan synthase alpha chain and this product enters the tryptophan metabolic pathway. It is well known that L-tryptophan is an aromatic amino acid and a biosynthetic precursor of microbial and host metabolites (69, 70). In this study, L-tryptophan generates a series of indole derivatives, including 3-Indoleacetic Acid (IAA), 5-methoxyindoleacetate and indole-3-acetamide. Interestingly, many microorganisms have been shown to synthesize IAA *via* the indole-3-acetamide and indolepyruvate pathways (71, 72). As a marker of gut microbiota metabolic activity (73), IAA plays a role in gut barrier integrity, immune cell activity and maintaining gut homeostasis (74, 75). Consistent with these findings, our data suggest that MOP is effective in increasing serum concentrations of indole derivatives in colitis and is significantly associated with gut microbes. This may be regulated by the metabolism of tryptophan by gut microbes. Interestingly, we found an interesting phenomenon that serotonin (5-hydroxytryptamine [5-HT]) and its derivative N-acetyl serotonin, produced by some gut microbes, were significantly enriched in the DSS+H MOP group, these signaling molecules are thought to regulate intestinal motility (76). It is worth noting that intestinal peristalsis dysfunction is a common symptom in patients with inflammatory bowel disease (77). Therefore, whether MOP can affect intestinal peristalsis through the intestinal microbial metabolite 5-HT to improve ulcerative colitis warrants future investigation.

In conclusion, we hypothesized that gut microbiota imbalance in UC mice causes abnormal lipid and amino acid metabolism, promoting gut barrier disruption. MOP may improve intestinal barrier integrity and improve colitis by regulating the disturbance of gut microbes and their metabolites (including lipid and amino acid metabolism).

## Materials and Methods

### Preparation of the *Moringa oleifera* Peptide (MOP) in Seed

The peptide (KETTTIVR) was synthesized (by GUOPING Pharmaceutical, Anhui, China) using a solid phase procedure that relied on Fmoc-protected amino acid synthesis methods (78). The peptide’s purity was > 95%, and the MOP formally identified by GUOPING Pharmaceutical (**Supplementary File 1: Supplementary Figure 1**).

### Animal Experiments

The animal protocol used in this study was reviewed and approved by the Institutional Animal Care and Use Committee of Yunnan Agricultural University (Yunnan, China) for ethical issues and scientific care [license no. SYXK (Dian) K2020-0006]. Six-week-old male C57Bl/6J mice (each 15–18 g) were obtained from Hunan SJA Laboratory Animal Co., Ltd., China [license no. SCXK (Hunan) 2019-0004]. Prior to the experiments, the mice were housed in a climate-controlled rearing box at 55% ± 5% relative humidity with a 12-h day/night cycle. After 1 week of acclimatization, the mice were randomly divided into five groups of 8 mice each: control, DSS, DSS+200 mg/kg MOP (DSS+L MOP), DSS+400 mg/kg MOP (DSS+M MOP), and DSS +800 mg/kg MOP (DSS +H MOP) (**Figure 1A**). The UC model was induced by administering to mice 3% (w/v) DSS (molecular weight 36,000–50,000 kDa; MP Biomedicals, UK) in their drinking water for 10 days. The MOP was dissolved in water. At the same time, the mice in the control group and the DSS group were gavaged with an equal volume of water. Body weight and disease activity index (DAI), including stool consistency and rectal bleeding, were monitored daily.

### Histology and Immunohistochemistry

Fresh colon tissues were fixed in 4% paraformaldehyde solution (Sigma-Aldrich), embedded in paraffin, sectioned (3-μm thickness), and stained with hematoxylin and eosin (H&E) and Alcian Blue. Tissue sections were incubated with Ly6G (Abcam, ab25377) and F4/80 (Abcam, ab6640). Images of each were collected using a microscope (Olympus, Tokyo, Japan) and then analyzed with Image pro-Plus 6.0 software (Media Cybernetics, Inc., MD, USA), and colitis was scored histologically as previously described (79). Crypt depth, goblet cells, Ly6G and F4/80 positive cells were counted.

### Evaluation of Serum Cytokine and Myeloperoxidase Activity

Cytokine levels and myeloperoxidase (MPO) activity in serum were determined using enzyme-linked immunosorbent assay (ELISA) kits, by following the manufacturer’s manual (Meimian, Jiangsu, China) instructions.

### RT-qPCR

Total RNA extractions from colon tissue samples were performed and reverse transcribed. Gene expression was measured using the SYBR Realtime PCR Kit (Takara, Japan) in the LightCycler/LightCycler 480 System (Roche Diagnostics, USA). *RPL-19* served as the internal reference. The mean Ct values of triplicate analyses were normalized from the mean Ct values of *RPL-19*. Primer sequences were described and synthesized by Generay Biotech Co., Ltd (Shanghai, China) (**Supplementary Table S2**).

### Western Blotting Analysis

Mouse colon tissues were excised and their protein concentrations determined. Proteins were separated by SDS-polyacrylamide gel electrophoresis, and then transferred onto polyvinylidene fluoride (PVDF) membranes (0.45 μm; Millipore, USA). To these, specific antibodies (ZO-1 (Abcam, ab190085), Occludin (Abcam, ab216327), Claudin1 (Abcam, ab180158), JAK2 (Abcam, ab108596), pJAK2 (Abcam, ab32101), STAT3 (Abcam, ab68153), pSTAT3 (Abcam, ab267373) and β-Tubulin (Abcam, ab18207) were added for the immunoreaction, with the chemiluminescence reaction performed to observe the protein bands. β-tubulin was used as an internal control. Image J software was used to analyze the gray value of obtained protein bands.

### RNA Sequencing

Total RNA was extracted from colon tissue samples according the manufacturer’s instructions (Invitrogen). Then RNA quality was determined by 2100 Bioanalyser (Agilent) and quantified using the ND-2000 (NanoDrop Technologies). RNA-seq transcriptome library was prepared following TruSeq™ RNA sample preparation Kit from Illumina (San Diego, CA) using RNA samples with high -quality. Paired-end RNA-seq sequencing library was sequenced with the Illumina HiSeq xten/NovaSeq 6000 sequencer.

### Sequencing of 16S rRNA Genes of Gut Microbiota

Total microbial genomic DNA was extracted from mouse colon contents. The quality and concentration of DNA were determined by agarose gel electrophoresis and a NanoDrop^®^ ND-2000 spectrophotometer (Thermo Scientific Inc., USA). PCR amplifications were performed, and their ensuing products were extracted and purified. Purified amplicons were pooled in equimolar amounts and paired on the Illumina MiSeq PE300 platform/NovaSeq PE250 platform (Illumina, San Diego, USA), this done according to the standard protocol of Majorbio Bio-Pharm Technology Co. Ltd. (Shanghai, China) End Sequencing.

### Non-Targeted Metabolomics

Serum samples from mice were extracted and transferred to a sample vial with an inner cannula for its machine analysis carried out using the UPLC-TripleTOF System of AB SCIEX. The resulting LC-MS raw data were processed using the metabolomics software program Progenesis QI (Waters Corporation, Milford, USA), to obtain the final data matrix for use in the formal analysis. Meanwhile, their MS and MSMS mass spectral information was searched against and matched with that in two public metabolic databases, HMDB (http://www.hmdb.ca/) and Metlin (https://metlin.scripps.edu/), to obtain each metabolite’s information.

## Data Availability Statement

The sequences generated in this study are stored in the National Center for Biotechnology Information (NCBI) and the project numbers are PRJNA822105 and PRJNA824237. The raw spectral data for metabolome analysis are freely available at https://data.mendeley.com/datasets/rsn78z2bnp/1. Additionally, all other data is contained within the article and **Supplementary Material**.

## Ethics Statement

The animal study was reviewed and approved by Institutional Animal Care and Use Committee of Yunnan Agricultural University.

## Author Contributions

JS and YT conceived and designed the experiments. Z-SH, JX, X-FW, J-JD, J-YM, Y-YB and YT performed the experiments. YT, JX, and Z-SH analyzed the data. JS wrote the paper. All authors reviewed the manuscript. All authors contributed to the article and approved the submitted version.

## Funding

This work was supported by Major Project of Science and Technology Department of Yunnan Province (202002AA100005 and 202102AE090027-2), YEFICRC project of Yunnan provincial key programs (2019ZG009), Cassava Industrial Technology System of China (CARS-11-YNSJ), Industrial Technology Leading Talent Program of the Ten Thousand People of Yunnan Province(A3032021174), and Yunnan Province Young and Middle-aged Academic and Technical Leaders Reserve Talents Project (2018HB040).

## Conflict of Interest

The authors declare that the research was conducted in the absence of any commercial or financial relationships that could be construed as a potential conflict of interest.

## Publisher’s Note

All claims expressed in this article are solely those of the authors and do not necessarily represent those of their affiliated organizations, or those of the publisher, the editors and the reviewers. Any product that may be evaluated in this article, or claim that may be made by its manufacturer, is not guaranteed or endorsed by the publisher.
