# Supplementary figures and images for: Moringa oleifera Lam. Peptide Remodels Intestinal Mucosal Barrier by Inhibiting JAK-STAT Activation and Modulating Gut Microbiota in Colitis
Source: Front Immunol. 2022 Jul 15;13:924178. doi: 10.3389/fimmu.2022.924178 (PMC9336532; doi:10.3389/fimmu.2022.924178)

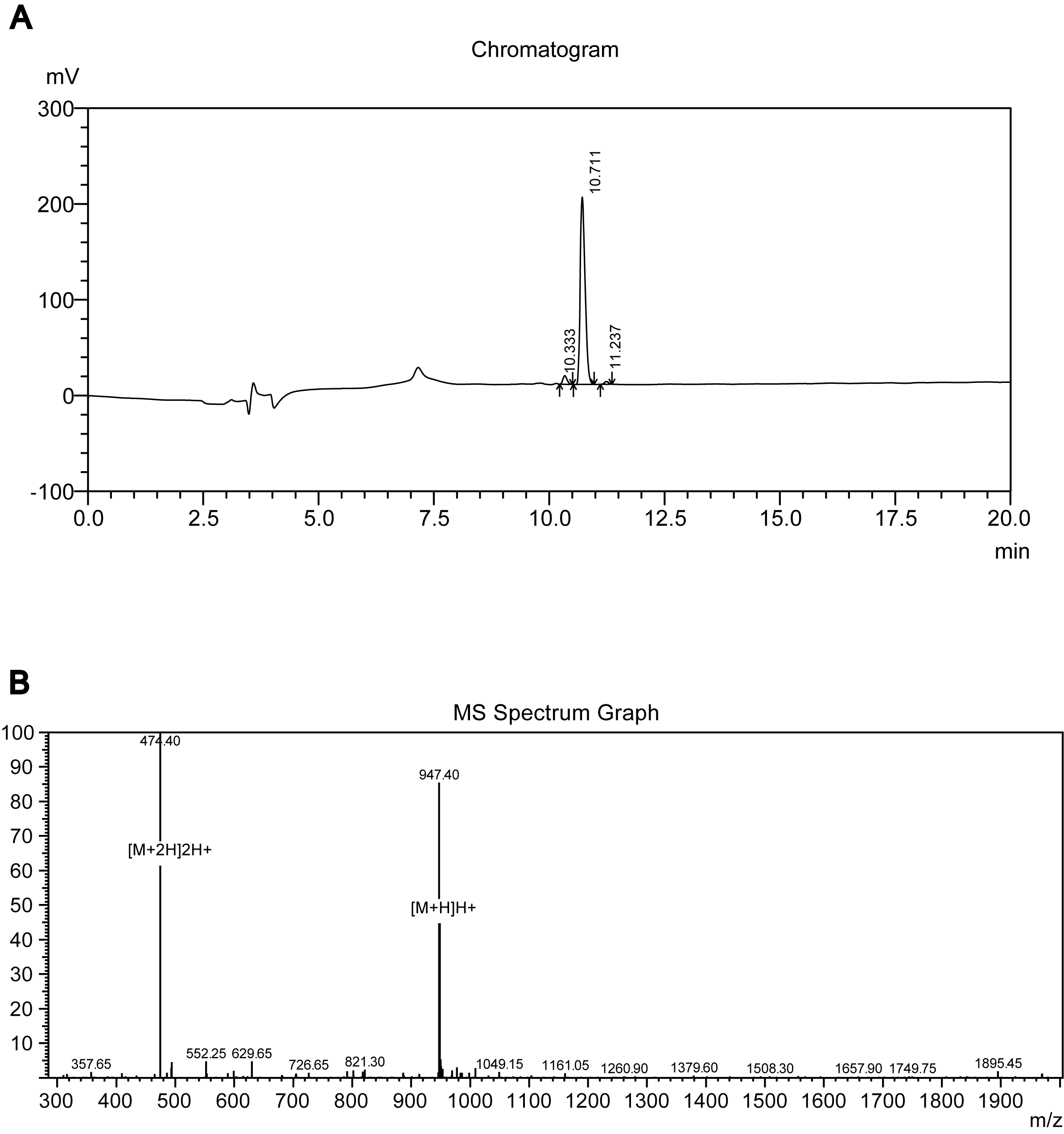

Supplement: Supplementary file 1 [file Image_1.tif]

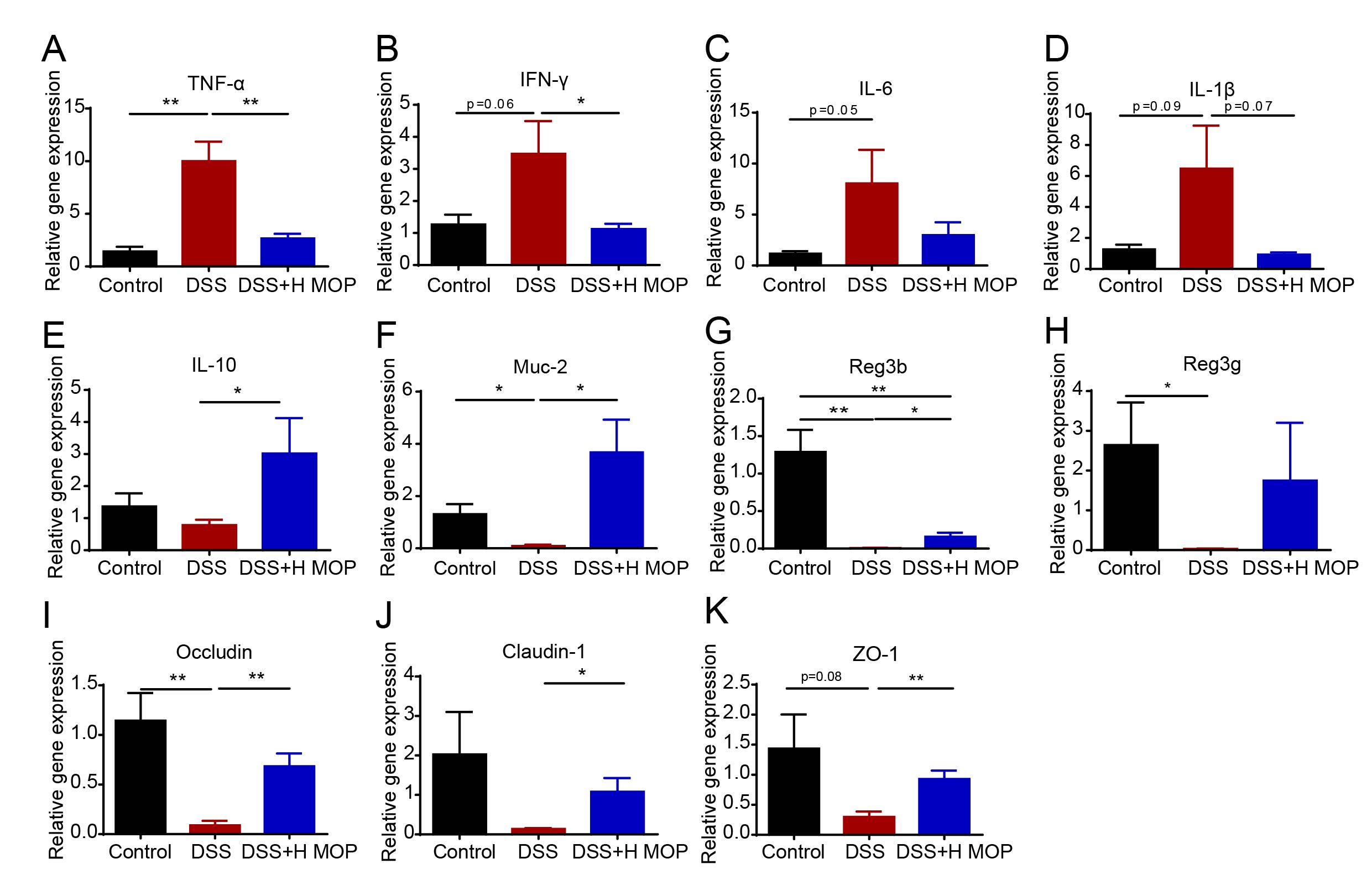

Supplement: Supplementary file 2 [file Image_2.jpeg]

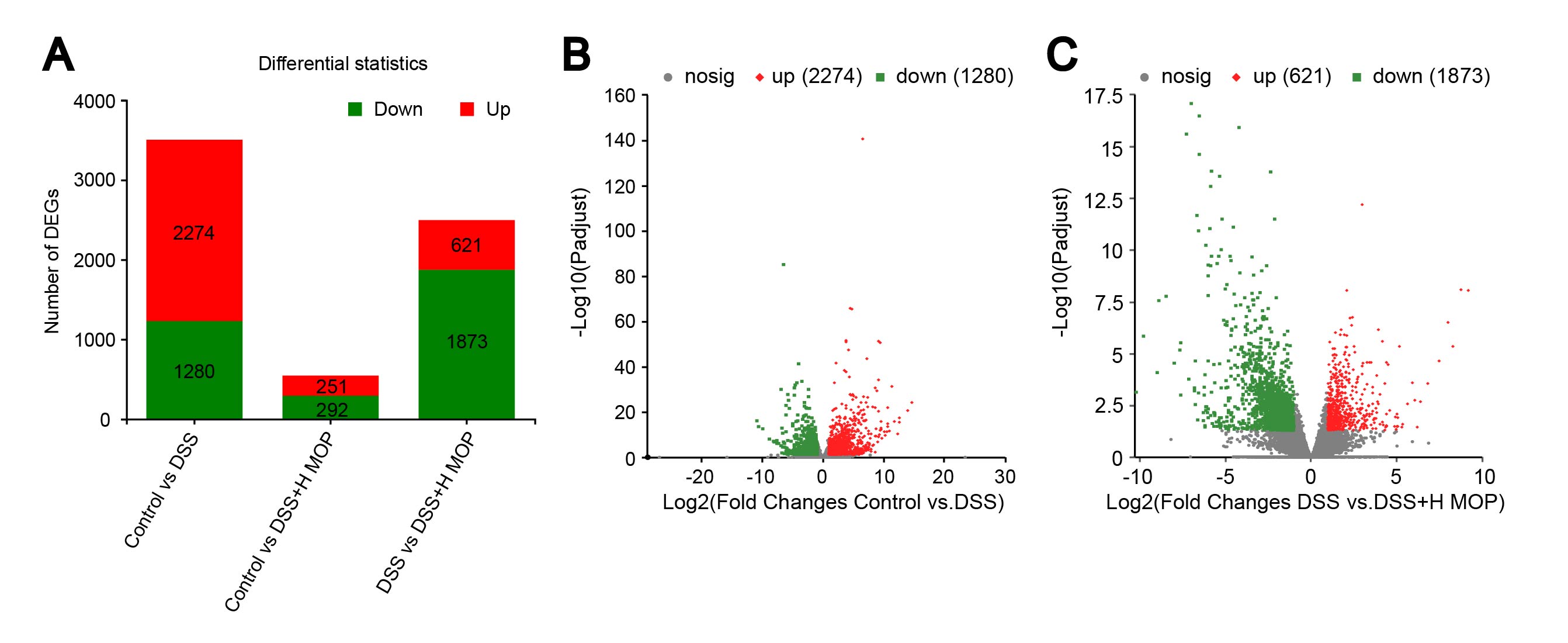

Supplement: Supplementary file 3 [file Image_3.jpeg]

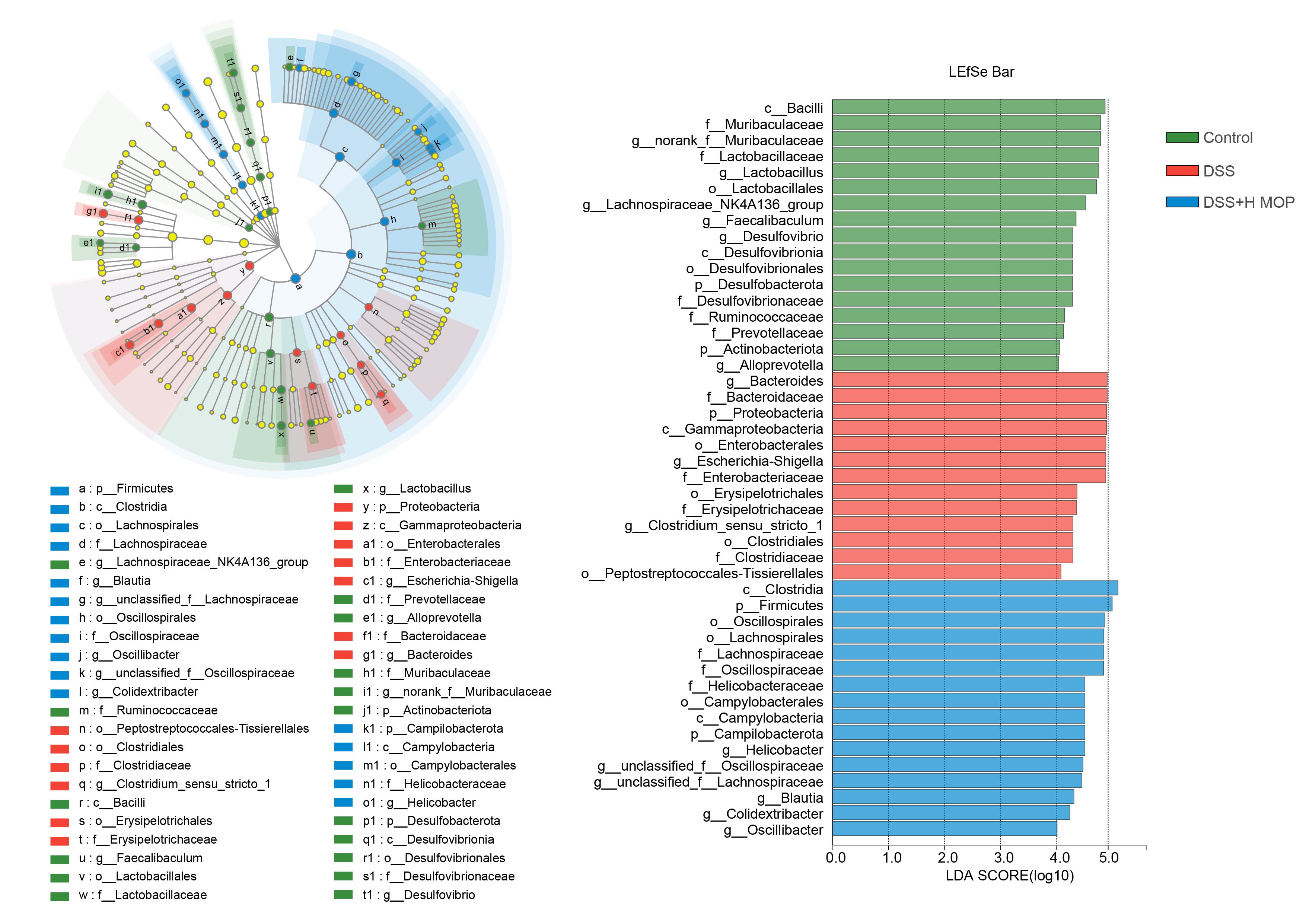

Supplement: Supplementary file 4 [file Image_4.jpeg]

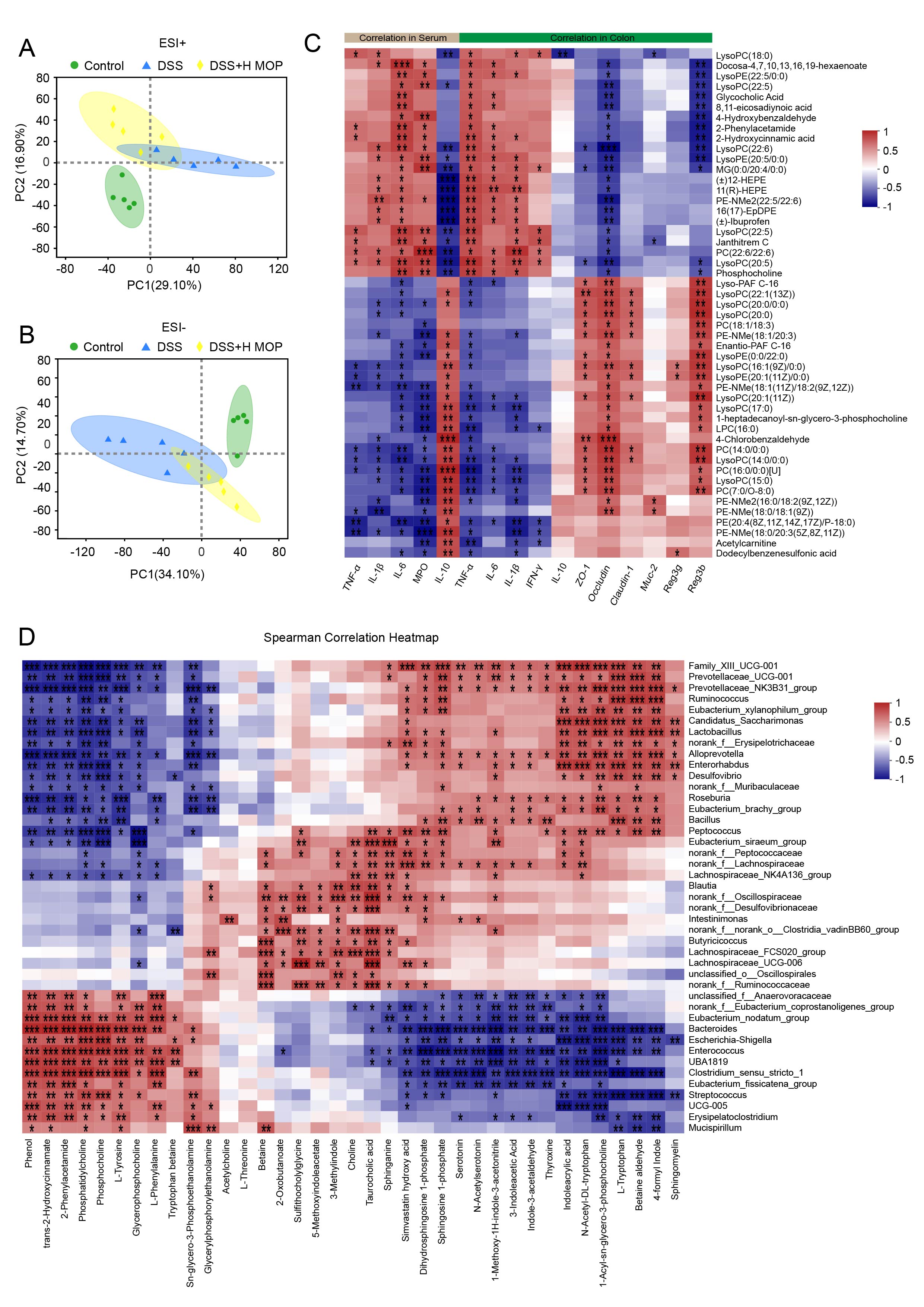

Supplement: Supplementary file 5 [file Image_5.jpeg]
