# Supplementary material for: Moringa oleifera Lam. Peptide Remodels Intestinal Mucosal Barrier by Inhibiting JAK-STAT Activation and Modulating Gut Microbiota in Colitis
Source: Front Immunol. 2022 Jul 15;13:924178. doi: 10.3389/fimmu.2022.924178 (PMC9336532; doi:10.3389/fimmu.2022.924178)
Supplement: Supplementary file 7 [file Table_2.docx]

Supplementary Table S2. Mice primer sequences in RT- PCR assays.

| **Genes** | **Forward** | **Reverse** |
| --- | --- | --- |
| TNF-α | AGACCCTCACACTCAGATCA | TCTTTGAGATCCATGCCGTTG |
| INF-γ | ATCTGGAGGAACTGGCAAAA | TTCAAGACTTCAAAGAGTCTGAG |
| IL-1β | TCCATGAGCTTTGTACAAGGA | AGCCCATACTTTAGGAAGACA |
| IL-6 | GTTCTCTGGGAAATCGTGGA | TGTACTCCAGGTAGCTA |
| IL-10 | AAGGACCAGCTGGACAACAT | TCTCACCCAGGGAATTCAAA |
| Occludin | ATGTCCGGCCGATGCTCTC | TTTGGCTGCTCTTGGGTCTGTAT |
| ZO-1 | TTTTTGACAGGGGGAGTGG | TGCTGCAGAGGTCAAAGTTCAAG |
| Claudin-1 | TGCCCCAGTGGAAGATTTACT | CTTTGCGAAACGCAGGACAT |
| Muc2 | ACGTGTCATATTTGCACCTCT | TCAACATTGAGAGTGCCAACT |
| Reg3b | CCCAGGCTTATGGCTCCTAC | ATGGAGCCCAATCCAAGTGT |
| Reg3g | TTCCTGTCCTCCATGATCAAA | CATCCACCTCTGTTGGGTTC |
| Osm | CAGAATCAGGCGAACCTCACG | AGCTCTCAGGTCAGGTGTGTT |
| IL5ra | CGTGTCCACCTCCCGTATCTA | AGACATACCTCTCCCTGGAGT |
| IL-21r | CTCACTTGCTACACTGACTACCT | GACTGGTCCGTCACATTGACA |
| Csf3 | ATGGCTCAACTTTCTGCCCAG | CTGACAGTGACCAGGGGAAC |
| Stat3 | CACCTTGGATTGAGAGTCAAGAC | AGGAATCGGCTATATTGCTGGT |
| IL4ra | ACACTACAGGCTGATGTTCTTCG | TGGACCGGCCTATTCATTTCC |
| Stat4 | TGGCAACAATTCTGCTTCAAAAC | GAGGTCCCTGGATAGGCATGT |
| Bcl2 | GAGAGCGTCAACAGGGAGATG | CCAGCCTCCGTTATCCTGGA |
| IL22ra2 | CATTGCCTTCTAGGTCTCCTCA | CCTGCTTGCCAGTGCAAAAT |
| RPL-19 | GAAGGTCAAAGGGAATGTGTTCA | CCTTGTCTGCCTTCAGCTTGT |
